# Art therapy is associated with sustained improvement in cognitive function in the elderly with mild neurocognitive disorder: findings from a pilot randomized controlled trial for art therapy and music reminiscence activity versus usual care

**DOI:** 10.1186/s13063-018-2988-6

**Published:** 2018-11-09

**Authors:** Rathi Mahendran, Mihir Gandhi, Rajesh Babu Moorakonda, Jonathan Wong, Madhu Mathi Kanchi, Johnson Fam, Iris Rawtaer, Alan Prem Kumar, Lei Feng, Ee Heok Kua

**Affiliations:** 1 Department of Psychological Medicine, Yong Loo Lin School of Medicine, National University of Singapore, NUHS Tower Block, Level 9, 1E Kent Ridge Road, Singapore, 119228 Singapore; 2 Department of Psychological Medicine, National University Hospital, NUHS Tower Block, Level 9, 1E Kent Ridge Road, Singapore, Singapore; 3 Academic Development Department, Duke-NUS Medical School, 8 College Road, Singapore, Singapore; 4 Department of Biostatistics, Singapore Clinical Research Institute, 31 Biopolis Way, Singapore, Singapore; 5 Centre of Quantitative Medicine, Duke-NUS Medical School, 8 College Road, Singapore, Singapore; 6 Tampere Center for Child Health Research, University of Tampere and Tampere University Hospital, Tampere, Finland; 7 Department of Psychiatry, Sengkang General Hospital, 110 Sengkang East Way, Singapore, Singapore; 8 Cancer Science Institute of Singapore, National University of Singapore, 14 Medical Drive, Singapore, Singapore; 9 Medical Science Cluster, Yong Loo Lin School of Medicine, National University of Singapore, Singapore, Singapore

**Keywords:** Art therapy, Music reminiscence activity, Elderly, Mild cognitive impairment

## Abstract

**Background:**

Mild cognitive impairment (MCI) is a phase in cognitive decline when it is still possible to intervene to reverse the decline. Cognitive stimulation delivered through psychosocial interventions provides both psychological intervention and social stimulation to improve cognition. A pilot open-label parallel-arms randomized controlled trial was undertaken to examine the effects of art therapy (AT) and music reminiscence activity (MRA) compared to the control, on the primary outcome of neurocognitive domain assessments in elderly people with MCI.

**Methods:**

Community-living elderly people with MCI (Petersen’s criteria), assessed for study eligibility, were randomized using a web-based system with equal allocation to two intervention arms: AT (guided viewing of art pieces and production of visual arts) and MRA (listening, and recalling memories related to music) and a control arm (standard care without any intervention). Interventions were led by trained therapists weekly for 3 months, then fortnightly for 6 months. Neurocognitive domains (mean of memory, attention, and visuo-spatial abilities standardized scores), psychological wellbeing (subsyndromal depression and anxiety) and telomere length as a biological marker of cellular ageing, were assessed by intervention-blinded assessors at baseline, 3 months and 9 months.

**Results:**

In total, 250 people were screened and 68 were randomized and included in the analysis. In the AT arm, neurocognitive domains improved compared to the control arm at 3 months (mean difference (d) = 0.40; 90% CI 0.126, 0.679) and were sustained at 9 months (d = 0.31; 90% CI 0.068, 0.548). There was some improvement in depression and anxiety at 3 and 9 months and in telomere length at 9 months, but this was not significant. Similar improvements were observed in the MRA arm over the control arm, but they were not significant. There were no intervention-related adverse effects.

**Conclusions:**

Art therapy delivered by trained staff as “art as therapy” and “art psychotherapy” may have been the significant contributor to cognitive improvements. The findings support cognitive stimulation for elderly people with cognitive decline and signal the need for larger studies and further investigation of carefully designed psycho-social interventions for this group.

**Trial registration:**

Clinical Trials.gov, NCT02854085. Registered on 7 July 2016.

**Electronic supplementary material:**

The online version of this article (10.1186/s13063-018-2988-6) contains supplementary material, which is available to authorized users.

## Background

Psycho-social interventions are increasingly investigated as preventive strategies for elderly people with cognitive decline. To date, there is little evidence of benefit when dementia (or major neurocognitive disorder, *Diagnostic and statistical manual of mental disorders* (DSM)-5) has set in when even cognitive training and cognitive stimulation interventions cannot significantly improve general cognition [1, 2]. Although attention has shifted to pre-dementia or mild cognitive impairment (MCI) (mild neurocognitive disorder, DSM-5), there is again no evidence that any intervention is effective at this stage and furthermore, pharmacotherapy is not recommended for MCI [3, 4]. The elderly with MCI are unfortunately a particularly vulnerable “at-risk” group. Almost half will deteriorate to dementia and at a higher rate than those who are cognitively normal [5, 6]. Up to 40% who may remain at the MCI stage, will continue to experience cognitive difficulties and psychological sequelae [7]. Interventions that improve domains of memory and executive function would therefore be particularly beneficial if they could contribute to a slowing of the progression or even a reversal of cognitive impairment.

In an earlier naturalistic study, Rawtaer et al*.* [8] found improvements in subsyndromal anxiety and depression amongst community-living elderly who participated in psychosocial interventions, particularly mindful awareness practice, art therapy (AT) and music reminiscence activity (MRA). However, when the literature on these psychosocial interventions was explored for cognitive effects in elderly people with MCI, it was evident that elderly populations with MCI have not been studied, many of the studies on AT and MRA interventions were not randomized, and interventions were mainly activities like art and craftwork or listening to music, rather than therapy. Thus, to examine the effects of both these interventions on cognitive functions in elderly people with MCI, the interventions were carefully reviewed, designed and structured as therapy, and a pilot randomized controlled trial (RCT) was undertaken. The primary findings of the effects on cognition, subsyndromal mood states and telomere lengths are presented. The primary objective was the comparison of change in neuropsychological test scores at 3 months between the intervention and control groups; the secondary objectives were comparisons at 9 months.

## Methods

### Participants and study design

An open-label, parallel RCT with three arms (two interventions and one control) recruited community-living elderly people who met pre-defined inclusion criteria, (age 60–85 years, both genders, community-living, fulfill Petersen’s criteria for MCI) [9]. The study had ethics approval from the National University of Singapore Institutional Review Board and written informed consent was taken from participants. Subjects who had been in a large cohort study at the University Department’s research site in the community, (known as the Training and Research Academy, TaRA) and who had given consent to be approached for intervention studies were contacted and provided information about the study. Recruitment, screening and informed consent and subsequent interventions and assessments were done at TaRA. Interventions were administered weekly in the first 3 months, then fortnightly for 6 months. Participants were randomly allocated into three arms (1:1:1 allocation) using a web-based randomization system, provided by the Singapore Clinical Research Institute, and intervention assignment was balanced using the permuted block randomization stratified by gender. A detailed study protocol was previously published [10].

### Interventions

AT involved two components. Art pieces were selected by curators from the National Gallery and the National University of Singapore Museum and the activity was developed in consultation with the study team (psychiatrists and therapists), with emphasis on relevance to the elderly, in terms of themes and events from the country’s past. Guided viewing and cognitive evaluation of art works at the respective sites was conducted as a group activity by trained staff and involved narration of thoughts and inner experiences. A second component involved visual art production. The physical creation of themed artwork was followed by image appreciation activities to gain insight and discuss feelings, and took place at the research center. MRA involved listening, and recalling memories and experiences related to the music. The therapist prepared songs and used photographs or video clips to accompany the music, for discussion purposes. The MRA promoted shared feelings while the group process provided validation. The detailed contents of the structured interventions are available in the study protocol [10]. Further details on the art works used and sample art works created by the participants will be available on request. The control group (CG) did not receive any intervention but continued life as usual.

### Outcome measures

Neurocognitive domain assessments at baseline and at 3 and 9 months reported here include the Rey auditory verbal learning test (RAVLT) List Learning, Delayed Recall, Recognition Trial (Memory), Wechsler Adult Intelligence Scale-3rd edition (WAIS-III) Block design (Visuospatial abilities), Digit Span Forward (Attention and Working Memory), and Color Trails Test 2 (Executive function) [11–13]. Individual domain scores were standardized (z-scores) to general population norms adjusted for age and education level; higher scores indicate better performance [14].

Psychological wellbeing was assessed at baseline and at 3 and 9 months with the (1) Geriatric Depression Scale (GDS), which is a 15-item “yes/no” questionnaire with higher total scores associated with higher risk of depression [15] and (2) Geriatric Anxiety Inventory (GAI), which is a 20-item “agree/disagree” questionnaire measuring dimensional anxiety, with higher total scores associated with anxiety symptoms [16].

Sleep quality was assessed using a 100-point visual analog scale (VAS) (0, worst sleep to 100, best sleep).

For telomere length measurements, genomic DNA was extracted from whole blood samples using QIA amp DNA blood mini kit (catalog number 51104) according to the manufacturer’s protocol and stored at − 80 °C. Telomere length was measured by using a non-radioactive chemiluminescent telomere length assay kit (Telo TAGGG assay kit, Sigma Aldrich; catalog number 12209136001) to visualize the telomeric DNA repeat sequence TTAAGGG from blood samples. Telomere length measurement involves the digestion of 1 μg of DNA using Hinf I/Rsa I enzymes at 37 °C for 2 h, and run on 0.8% agarose gel. DNA smears were transferred on to the nylon membrane (Amersham Hybond ™-XL) overnight. Transferred DNA fragments are hybridized to a digoxigenin (DIG)-labeled probe to validate the telomeric repeats developed by CDP-Star which is a digoxigenin substrate to capture the imaging on x-ray film. Telomere length was measured by the location of bands based on molecular weight standard. Average telomere length is measured between 100 base pairs to 20 k base pairs. TeloTool, which is MATLAB software, was used to measure the telomere lengths of the samples in this study. Image processing and detection of DNA smears were evaluated as indicated in the references [17, 18].

### Statistical analysis

The sample size of the study was calculated to estimate the intervention effect (i.e., difference between the intervention and control group for mean change in neuropsychological test score at 3 months from baseline) with pre-specified precision. A sample size of 22 participants in each group will provide an estimate with precision +/− 0.5 standard deviation (SD) with 90% certainty (i.e., width of 90% confidence interval (upper limit – lower limit) equal to 1 SD). The precision level was selected considering an intervention will be worth investigating further in a confirmatory trial if at the least a medium standardized effect size (0.5 SD) is shown in comparison to the control group.

Mean change from baseline in neurocognitive z-scores were estimated and compared between the interventions and the control groups using the linear mixed model with participant-specific random effects and indicator variables for the two interventions (reference, control group), indicator variables for time (3-month and 9-month assessments with baseline as the reference), interactions between indicator variables of the interventions and time, baseline value, and gender as fixed effects, along with 90% and 95% CIs. No multiplicity corrections were applied due to the exploratory nature of the study. The same model was used to compare the Geriatric Depression Scale (GDS) total score, Geriatric Anxiety Index (GAI) total score, sleep quality VAS, and telomere length between the interventions and the control groups. All the analyses were performed in the intention-to-treat population. SAS software version 9.4 (SAS Institute, Cary, NC, USA) was used. A *p* value <0.05 was deemed to be statistically significant: *p* values should be interpreted as hypothesis generating as the study was not powered for hypothesis testing.

## Results

### Participant characteristics and study conduct

Between 13 June 2016 and 17 August 2016, 250 potential participants were screened of whom 68 were recruited into the study, randomly assigned to AT (*n* = 22), MRA (*n* = 24), or the CG (*n* = 22), and included in the intention-to-treat population. There were no clinically relevant differences among the three groups at baseline (Table 1), except the CG had a larger percentage of participants (41%) who were currently working (full time/part-time/self-employed) compared to the AT (18%) and (8%) MRA groups. Overall, the mean age of the participants was 71.1 years, 56 participants were female, and the average duration of schooling was 5.4 years.

**Table 1:** Baseline demographic and neurocognitive characteristics

Characteristics	Art therapy (*n* = 22)	Music reminiscence activities (*n* = 24)	Control (*n* = 22)
Age (years), mean (SD)	71.1 (4.8)	71.6 (5.3)	70.6 (5.8)
Female, *n* (%)	18 (81.8)	20 (83.3)	18 (81.8)
Living with partner, *n* (%)	14 (63.6)	15 (62.5)	20 (90.9)
Education years, mean (SD)	5.2 (4.1)	5.0 (3.3)	6.1 (3.4)
Working, *n* (%)	4 (18.2)	2 (8.3)	9 (40.9)
Body mass index, mean (SD)	24.9 (3.6)	23.5 (3.8)	22.8 (3.9)
Medical condition, *n* (%)			
High blood pressure	14 (63.6)	18 (75.0)	6 (27.3)
High cholesterol	11 (50.0)	15 (62.5)	7 (31.8)
Diabetes mellitus	2 (9.1)	8 (33.3)	5 (22.7)
Others	2 (9.1)	3 (12.5)	3 (13.6)
Neurocognitive domain z-score, mean (SD)			
RAVLT List Learning Sum	0.04 (1.35)	0.01 (1.26)	−0.01 (1.26)
RAVLT Delayed Recall	−0.05 (0.98)	− 0.01 (1.10)	0.05 (0.87)
RAVLT Recognition Trial	−0.05 (1.28)	0.26 (1.10)	0.14 (0.91)
Mean of memory domains (RAVLT subsets)	−0.02 (0.97)	0.09 (0.95)	0.06 (0.79)
WAIS-III Block Design	−0.74 (1.16)	−0.93 (1.04)	− 0.47 (0.85)
WAIS-III Digit Span (Forward)	1.33 (1.59)	1.79 (1.65)	2.15 (1.77)
Color Trails 2	−0.55 (1.38)	−1.15 (1.46)	−0.82 (1.91)
Mean of all domains	−0.00 (0.42)	−0.01 (0.59)	0.17 (0.56)
Number of domains with z-score < − 1.5, mean (SD)	1.41 (0.80)	1.71 (0.81)	1.14 (0.35)
GAI total score, mean (SD)	2.0 (3.9)	2.1 (4.0)	2.8 (4.1)
GAD total score, mean (SD)	2.5 (2.9)	1.8 (2.2)	2.8 (2.9)
Sleep quality VAS, mean (SD)	67.0 (26.1)	68.4 (19.8)	69.8 (16.5)

See “Methods” section for outcome definitions

*RAVLT* Rey Auditory Verbal Learning Test, *WAIS-III* Wechsler Adult Intelligence Scale-3rd edition, *GAI* Geriatric Anxiety Inventory, *GAD* Geriatric Depression Scale, *SD* standard deviation

**Fig. 1:**
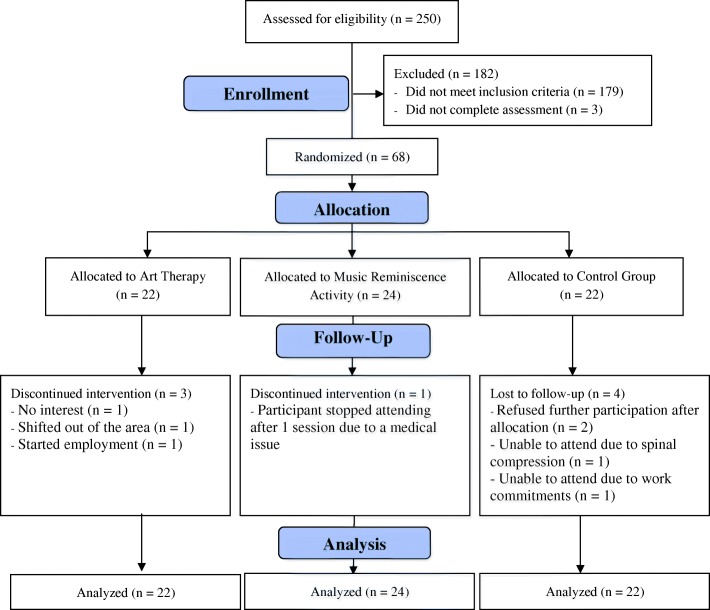
Participants disposition (Consolidated Standards of Reporting Trials (CONSORT) diagram)

During the first 3 months post-randomization, three participants from the AT group, one from the MRA group, and four from the CG group discontinued the study (Fig. 1). Mean compliance (attendance at intervention therapy sessions) with AT and MRA was 80% and 82%, respectively. By the end of the study at 9 months, the AT group, MRA group, and CG had 18, 22, and 18 participants, respectively; mean compliance with the AT and MRA interventions was 75% and 83%, respectively.

Each of the intervention sessions (for AT and MRA) lasted an hour inclusive of a 5-min mindful relaxation exercise at the start, to settle the subjects, and a 15-min break). The 40 min of active engagement was effective and did not tire the elderly participants; they remained engaged throughout the session. No subject left any of the sessions before it ended. Travelling time by coach to the National Gallery or NUS Museum took about 15 min. The therapists, were advised on the participants’ educational level and cognitive state and were the same throughout the study period and every effort was made to ensure they understood the intervention and sessions.

### Changes in neuropsychological test scores

**Fig. 2:**
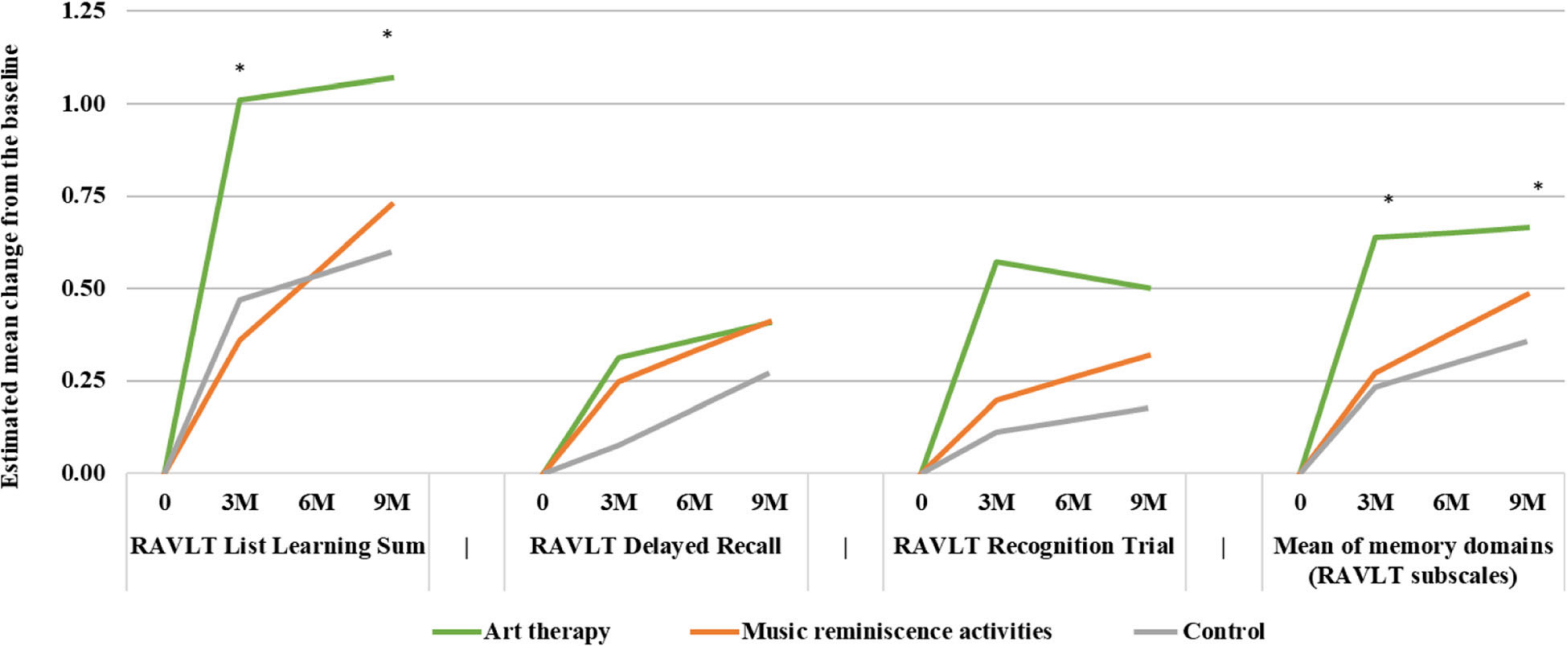
Estimated mean change in memory-related neuropsychological outcomes at 3 months (3M) and 9 months (9M) from baseline: *p < 0.05 for comparison with the control group. RAVLT, Rey Auditory Verbal Learning Test. Outcomes are presented as standardized z-score (see the “Methods” section). Mean values were estimated using a linear mixed-effects model adjusted for baseline values and gender

The estimated mean change at 3 months from baseline was statistically significantly higher in the AT group compared to the CG for List Learning (difference (d) = 0.542; 90% confidence interval (CI) 0.105, 0.810; *p* = 0.042) and Digit Span Forward (d = 0.991; 90% CI 0.251, 1.730; *p* = 0.028) (Fig. 2). Furthermore, Beneficial effects were also observed in the AT group for Delayed Recall, Recognition Trials, Block Design, and Color Trails 2; however, these results were not statistically significant (each *p* > 0.05). The mean of memory domains (List Learning, Delayed Recall, Recognition Trial) and the mean of all domains (List Learning, Delayed Recall, Recognition Trial, Digit Span Forward, Color Trains 2, Block Design) were also statistically significantly higher in the AT group compared to the CG (d (memory domains) = 0.403; 90% CI 0.126, 0.679; *p* = 0.017; d (all domains) = 0.462; 90% CI 0.202, 0.722; *p* = 0.004). Mean number of domains with z-score < − 1.5 at 3 months was lower in the AT group compared with the CG (d = − 0.314; 90% CI − 0.629, 0.000; *p* = 0.100). The effect of AT on memory domains was also sustained at 9 months (d = 0.308; 90% CI 0.068, 0.548; *p* = 0.035). The detailed results of memory-related and other neuropsychological outcomes are reported in Table 2 and Additional file 1: Tables S1 and Table S2, respectively.

**Table 2:** Estimated mean change in memory-related neuropsychological outcomes at 3-months and 9-months

	Art therapy (*n* = 22)	Music reminiscence activities (*n* = 24)	Control (*n* = 22)
Number of participants assessed at 3 months	19	23	18
Number of participants assessed at 9 months	18	22	18
RAVLT List Learning Sum z-score			
Mean change at 3 months from baseline (SE)	1.01 (0.19)	0.36 (0.17)	0.47 (0.19)
Difference (intervention – control) (90% CI) [*p*]	0.54 (0.105, 0.978) [0.042]	−0.11 (− 0.529, 0.314) [0.674]	
Mean change at 9 months from baseline (SE)	1.07 (0.17)	0.73 (0.15)	0.60 (0.16)
Difference (intervention – control) (90% CI) [*p*]	0.47 (0.089, 0.854) [0.043]	0.13 (− 0.240, 0.493) [0.569]	
RAVLT Delayed Recall z-score			
Mean change at 3 months from baseline (SE)	0.31 (0.14)	0.25 (0.13)	0.08 (0.14)
Difference (intervention – control) (90% CI) [*p*]	0.24 (−0.088, 0.561) [0.230]	0.17 (− 0.142, 0.486) [0.366]	
Mean change at 9 months from baseline (SE)	0.41 (0.13)	0.41 (0.12)	0.27 (0.12)
Difference (intervention – control) (90% CI) [*p*]	0.14 (−0.159, 0.430) [0.448]	0.14 (− 0.145, 0.421) [0.419]	
RAVLT Recognition Trial z-score			
Mean change at 3 months from baseline (SE)	0.57 (0.22)	0.20 (0.20)	0.11 (0.22)
Difference (intervention – control) (90% CI) [*p*]	0.46 (−0.045, 0.969) [0.134]	0.09 (− 0.402, 0.578) [0.767]	
Mean change at 9 months from baseline (SE)	0.50 (0.21)	0.32 (0.19)	0.18 (0.20)
Difference (intervention – control) (90% CI) [*p*]	0.32 (−0.153, 0.802) [0.262]	0.14 (− 0.318, 0.600) [0.612]	
Mean z-score for RAVLT memory domains			
Mean change at 3 months from baseline (SE)	0.64 (0.12)	0.27 (0.11)	0.23 (0.12)
Difference (intervention – control) (90% CI) [*p*]	0.40 (0.126, 0.679) [0.017]	0.04 (− 0.230, 0.304) [0.819]	
Mean change at 9 months from baseline (SE)	0.67 (0.10)	0.48 (0.10)	0.36 (0.10)
Difference (intervention – control) (90% CI) [*p*]	0.31 (0.068, 0.548) [0.035]	0.13 (− 0.102, 0.358) [0.358]	

Mean values were estimated using a linear mixed-effects model adjusted for baseline values and gender. See “Methods” section for outcome definitions

*RAVLT* Rey Auditory Verbal Learning Test, *SE* standard error, *CI* confidence intervals

The estimated mean changes were higher in the MRA group compared to the CG for Delayed Recall (d = 0.172), Recognition Trial (d = 0.088), Digit Span Forward (d = 0.787), Color Trails 2 (d = 0.033), and Block Design (d = 0.130), but they were not statistically significant (each *p* > 0.05) (Fig. 2). Similarly, the means of memory domains and all domains were higher in the MRA group compared to the CG group but were not statistically significant (d (memory domains) = 0.037; d (all domains) = 0.180; each *p* > 0.05). The effect in the MRA group at 9 months was similar to that at 3 months (Table 2; Additional file 1: Tables S1 and S2).

### Changes in subsyndromal depression and anxiety

There was some decline (less than 2 points) in the GDS and GAI total scores at 3 months and 9 months from baseline in both the AT and MRA groups. However, these reductions were not statistically significantly different from the ones observed in the CG (d < 1 point in both the GDS and GAI total scores at each timepoint; *p* > 0.05). (Table 3 and Additional file 1: Table S3).

**Table 3:** Estimated mean change in anxiety, depression, and sleep quality outcomes at 3-months and 9-months

	Art therapy (*n* = 22)	Music reminiscence activities (*n* = 24)	Control (*n* = 22)
Number of participants assessed at 3 months	19	23	18
Number of participants assessed at 9 months	18	22	18
Geriatric Anxiety Inventory total score			
Mean change at 3 months from baseline (SE)	− 0.45 (0.72)	−0.98 (0.66)	−1.25 (0.75)
Difference (intervention – control) (90% CI) [*p*]	0.80 (−0.92, 2.52) [0.444]	0.27 (− 1.39, 1.92) [0.791]	
Mean change at 9 months from baseline (SE)	−0.17 (0.71)	− 0.75 (0.65)	−0.05 (0.72)
Difference (intervention – control) (90% CI) [*p*]	−0.12 (− 1.79, 1.56) [0.909]	−0.70 (− 2.30, 0.91) [0.474]	
Geriatric depression scale total score			
Mean change at 3 months from baseline (SE)	− 0.76 (0.62)	−0.75 (0.57)	−1.22 (0.64)
Difference (intervention – control) (90% CI) [*p*]	0.46 (−1.02, 1.93) [0.610]	0.46 (− 0.96, 1.89) [0.590]	
Mean change at 9 months from baseline (SE)	−1.06 (0.62)	− 1.15 (0.57)	−0.47 (0.63)
Difference (intervention – control) (90% CI) [*p*]	−0.59 (− 2.05, 0.88) [0.509]	−0.67 (− 2.08, 0.74) [0.431]	
Sleep quality visual analog scale			
Mean change at 3 months from baseline (SE)	6.94 (4.39)	−2.09 (4.09)	3.56 (4.62)
Difference (intervention – control) (90% CI) [*p*]	3.39 (− 7.15, 13.92) [0.595]	−5.65 (− 15.85, 4.55) [0.361]	
Mean change at 9 months from baseline (SE)	3.44 (4.17)	4.51 (3.82)	2.06 (4.29)
Difference (intervention – control) (90% CI) [*p*]	1.39 (− 8.54, 11.32) [0.817]	2.46 (− 7.08, 11.99) [0.670]	

Mean values were estimated using a linear mixed-effects model adjusted for baseline values and gender. See “Methods” section for outcome definitions

*SE* standard error, *CI* confidence intervals

### Change in sleep quality

There was an improvement of 7 points and 3 points in the sleep quality VAS in the AT group at 3 months and 9 months. However, these improvements were not statistically significantly better than the changes observed in the CG (d < 4 points at each timepoint; *p* > 0.05). The MRA group also had sleep quality similar to the CG at post-baseline assessments (Table 3 and Additional file 1 Table S3).

### Changes in telomere lengths

There was an increase in the telomere length in the AT group at 9 months (mean change = 552; *p* = 0.003). However, the change was not statistically significantly different from the increase observed in the CG (d = − 22; *p* > 0.05). The MRA group also had an increase in the telemere length at 9 months (mean change = 292; *p* = 0.076), but this was not statistically significantly different from the CG (d = − 281; *p* > 0.05) (Table 4 and Additional file 1: Table S4). There were no intervention-related adverse effects reported in the trial.

**Table 4:** Estimated mean change in telomere length at 3 months and 9 months

	Art therapy (*n* = 22)	Music reminiscence activities (*n* = 24)	Control (*n* = 22)
Number of participants assessed at 3 months	19	23	NA
Number of participants assessed at 9 months	18	22	18
Mean change at 3 months from baseline (SE)	197 (197)	−41 (186)	NA
Mean change at 9 months from baseline (SE)	552 (177)	292 (163)	573 (182)
Difference (intervention – control) (90% CI) [*p*]	− 22 (− 444, 400) [0.932]	− 281 (− 686, 124) [0.252]	

Mean values were estimated using a linear mixed-effects model adjusted for baseline values and gender. See “Methods” section for outcome definitions

*NA* not applicable (telomere length was not collected in the control group at 3 months post baseline), *SE* standard error, *CI* confidence intervals

## Discussion

This study provides preliminary evidence that specific psychosocial interventions that are carefully structured and regularly delivered by trained staff can effectively improve cognitive function in specific domains in elderly people with MCI [4]. Compared to previous studies on AT and MRA, this RCT was methodologically rigorous and suggests that AT has a greater benefit than MRA. AT had more significant effects than MRA with improvements in memory, attention, visuo-spatial abilities and executive function at 3 months and which was sustained in the memory domain at 9 months. We posit that both the cognitive evaluation of art works and the physical creation of art pieces, followed by discussions that involve different cognitive processes, contributed to the improvements with AT (Lee R, Wong J, Wong LS, Gandhi M, Rawtaer I, Feng L, Kua EH, Mahendran R: Art therapy for the prevention of cognitive decline, in preparation). AT delivered as a combination technique of “art-as-therapy” and “art-psychotherapy” may have been the critical process that led to the cognitive improvements.

Research to date supports the impact of art on the brain with several psychological [19, 20] and physiological [21] processes involving the integration of sensory input, internal decision-making and emotional processing, and joint attention, with accompanying neuroanatomical changes in functional connectivity in the default mode network [22] and activation in regions such as the orbitofrontal cortex [23].

While studies suggest that telomere lengths respond to lifestyle and mindset via telomerase activity, (an intracellular enzyme with a RNA reverse transcriptase component that lengthens telomeres) [24], recent work reflects that oxidative stress and inflammation need to be reversed for lengthening to occur [25]. Short telomere length is associated with impaired cognitive performance [26]. The better cognitive scores associated with telomere lengthening suggests that the psychosocial interventions were effective in reversing cellular-level inflammatory mechanisms that contribute to cognitive decline [27].

The study challenges the view that interventions are ineffective in MCI and supports the use of “single” psychosocial interventions when multifaceted interventions are not readily available or possible [2]. The frequency of guided interventions at weekly intervals, was also sufficiently effective in addressing cognitive decline, when a previous study had suggested engagement in stimulating activities at least a twice a week to reduce the risk of dementia by 50% [28]. Finally, the short time frame in which cognitive improvements were noted in the cognitive domains, especially memory and executive function is particularly significant for patients with MCI or early dementia where early interventions could increase the chances of delaying or even reversing cognitive decline.

Of note are the improvements in the CG even though they were not offered any intervention and allowed to continue life as usual. While it may be argued that such improvements reflect a lack of specificity of the interventions, it must be noted that 40% of the CG returned to employment during the study duration, which was not disallowed in the study protocol, and which could have accounted for the improvement in cognition scores. Employment may have provided cognitive stimulation, and the increase in daily physical activity, likely contributed to the gains seen in the CG. But these gains were associated with a negative effect as their mood state was affected as reflected in the worsened subsyndromal anxiety scores in the CG.

There are some limitations in this study that need to be highlighted. As this was a pilot study, the sample size was small and not powered for hypothesis testing. Therefore, the *p* values should be interpreted with caution. The majority of study participants were female. A similar preponderance of female participants was noted in our other psychosocial intervention studies; it is because many men are still employed and unable to commit to participation in studies. Double blinding, while ideal, is not possible in a study of this nature. However, we ensured that assessors were blind to the study intervention arm and participants were also reminded not to discuss their intervention with the assessors. Although we excluded participants with depression and anxiety, it is still possible that the improvements in subsyndromal depression and subsyndromal anxiety could have had a small confounding effect on cognitive functioning. We also did not interview participants to assess their emotional states. Furthermore, it could be argued that the design of the AT intervention, with the two components, requires more active participation than the MRA. Finally, we need to consider whether socialization had an impact on the response to the interventions and it has been suggested that this is possible via mechanisms such as joint attention and theory of mind [21, 29]. Although this was not examined, and may not have made a significant contribution to the results as participants were independent and community-living elderly with no psychiatric illnesses, these areas nonetheless deserve further inquiry.

The feasibility of this trial was dependent on several factors the most important being the intervention design and the program delivery. The AT intervention was more than just a participative activity described in other studies but involved both art viewing and cognitive evaluation, and art creation with discussion, analysis, and self-evaluation. The well-developed intervention underwent several reviews by the curators, therapists, and study investigators and may not be readily delivered except by trained art therapists, which would be a limiting factor especially for larger sample sizes. Additionally, identifying elderly people with MCI requires both clinical evaluation and detailed neuropsychological testing. There were also several aspects of the study logistics that must be noted. Art pieces could not be removed from the National Gallery or NUS Museum and so participants had to go to these venues for the art viewing sessions. To ensure that the elderly would arrive safely and on time, transportation was arranged and staff accompanied them from the community Research Centre which added to study costs. Buy-in and support from the National Gallery and NUS Museum were crucial in undertaking the study [30, 31].

## Conclusions

While there are inherent issues in the study design, it would be very difficult to control for these in undertaking a study of this nature. Although these factors may limit the generalizability of this RCT, this pilot RCT supports the effectiveness of cognitive stimulation programs, in particular art therapy for elderly peopoe with mild cognitive impairment and highlights the need for further evaluation of these interventions and replication on a larger scale.

## Additional file

Additional file 1: **Table S1.** Estimated mean and change from baseline (95% confidence interval) in memory-related neuropsychological outcomes at 3 months and 9 months. **Table S2.** Estimated mean and change from the baseline (90% and 95% confidence intervals) in neuropsychological outcomes (other than memory-related) at 3 months and 9 months. **Table S3.** Estimated mean and mean change from baseline (95% confidence interval) in anxiety, depression and sleep quality outcomes at 3 months and 9 months. **Table S4.** Estimated mean change in telomere length at 3 months and 9 months. (DOCX 26 kb)

## Abbreviations

AT: Art therapy
CG: Control group
CI: Confidence interval
GDS: Geriatric Depression Scale
GIA: Geriatric Anxiety Index
MCI: Mild cognitive impairment
MRA: Music reminiscence activity
*p*: *P* value
RAVLT: Rey Auditory Verbal Learning Test
RCT: Randomized controlled trial
SD: Standard deviation
TaRA: Training and Research Academy
WAIS: Wechsler Adult Intelligence Scale
