# Supplementary material for: Art therapy is associated with sustained improvement in cognitive function in the elderly with mild neurocognitive disorder: findings from a pilot randomized controlled trial for art therapy and music reminiscence activity versus usual care
Source: Trials. 2018 Nov 9;19:615. doi: 10.1186/s13063-018-2988-6 (PMC6230219; doi:10.1186/s13063-018-2988-6)
Supplement: Supplementary file 1 — Table S1. Estimated mean and change from baseline (95% confidence interval) in memory-related neuropsychological outcomes at 3 months and 9 months. Table S2. Estimated mean and change from the baseline (90% and 95% confidence intervals) in neuropsychological outcomes (other than memory-related) at 3 months and 9 months. Table S3. Estimated mean and mean change from baseline (95% confidence interval) in anxiety, depression and sleep quality outcomes at 3 months and 9 months. Table S4. Estimated mean change in telomere length at 3 months and 9 months. (DOCX 26 kb) [file 13063_2018_2988_MOESM1_ESM.docx]

**Additional file 1**

**Table S1. Estimated mean and change from baseline (95% confidence interval) in memory-related neuropsychological outcomes at 3-months and 9-months**

|  | **Art therapy ( N = 22 )** | **Music reminiscence activities ( N = 24 )** | **Control ( N = 22 )** |
| --- | --- | --- | --- |
| RAVLT List Learning Sum Z-score |  |  |  |
| Mean at baseline (SE) | 0.06 (0.14) | 0.05 (0.13) | 0.05 (0.14) |
| Mean at 3 months (SE) | 1.07 (0.15) | 0.42 (0.14) | 0.52 (0.15) |
| Mean at 9 months (SE) | 1.13 (0.15) | 0.78 (0.14) | 0.65 (0.15) |
| Mean change at 3 months from baseline (SE) | 1.01 (0.19) | 0.36 (0.17) | 0.47 (0.19) |
| Difference [Intervention – Control] (95% CI) | 0.54 (0.021, 1.063) [0.042] | -0.11 (-0.61, 0.396) [0.674] |  |
| Mean change at 9 months from baseline (SE) | 1.07 (0.17) | 0.73 (0.15) | 0.60 (0.16) |
| Difference [Intervention – Control] (95% CI) [*p*] | 0.47 (0.015, 0.928) [0.043] | 0.13 (-0.312, 0.564) [0.569] |  |
| RAVLT Delayed Recall Z-score |  |  |  |
| Mean at baseline (SE) | -0.02 (0.10) | -0.02 (0.10) | -0.01 (0.10) |
| Mean at 3 months (SE) | 0.29 (0.11) | 0.23 (0.10) | 0.07 (0.11) |
| Mean at 9 months (SE) | 0.39 (0.11) | 0.39 (0.10) | 0.27 (0.11) |
| Mean change at 3 months from baseline (SE) | 0.31 (0.14) | 0.25 (0.13) | 0.08 (0.14) |
| Difference [Intervention – Control] (95% CI) [*p*] | 0.24 (-0.151, 0.624) [0.230] | 0.17 (-0.202, 0.546) [0.366] |  |
| Mean change at 9 months from baseline (SE) | 0.41 (0.13) | 0.41 (0.12) | 0.27 (0.12) |
| Difference [Intervention – Control] (95% CI) [*p*] | 0.14 (-0.216, 0.487) [0.448] | 0.14 (-0.200, 0.476) [0.419] |  |
| RAVLT Recognition Trial Z-score |  |  |  |
| Mean at baseline (SE) | -0.02 (0.15) | 0.15 (0.15) | 0.08 (0.15) |
| Mean at 3 months (SE) | 0.55 (0.16) | 0.34 (0.15) | 0.19 (0.17) |
| Mean at 9 months (SE) | 0.48 (0.17) | 0.46 (0.16) | 0.26 (0.16) |
| Mean change at 3 months from baseline (SE) | 0.57 (0.22) | 0.20 (0.20) | 0.11 (0.22) |
| Difference [Intervention – Control] (95% CI) [*p*] | 0.46 (-0.143, 1.067) [0.134] | 0.09 (-0.497, 0.673) [0.767] |  |
| Mean change at 9 months from baseline (SE) | 0.50 (0.21) | 0.32 (0.19) | 0.18 (0.20) |
| Difference [Intervention – Control] (95% CI) [*p*] | 0.32 (-0.246, 0.895) [0.262] | 0.14 (-0.408, 0.689) [0.612] |  |
| Mean Z-score for RAVLT memory domains |  |  |  |
| Mean at baseline (SE) | 0.01 (0.09) | 0.03 (0.09) | 0.02 (0.09) |
| Mean at 3 months (SE) | 0.64 (0.09) | 0.3 (0.09) | 0.26 (0.10) |
| Mean at 9 months (SE) | 0.67 (0.10) | 0.51 (0.09) | 0.38 (0.09) |
| Mean change at 3 months from baseline (SE) | 0.64 (0.12) | 0.27 (0.11) | 0.23 (0.12) |
| Difference [Intervention – Control] (95% CI) [*p*] | 0.40 (0.072, 0.733) [0.017] | 0.04 (-0.282, 0.356) [0.819] |  |
| Mean change at 9 months from baseline (SE) | 0.67 (0.10) | 0.48 (0.10) | 0.36 (0.10) |
| Difference [Intervention – Control] (95% CI) [*p*] | 0.31 (0.022, 0.595) [0.035] | 0.13 (-0.147, 0.403) [0.358] |  |

RAVLT: Rey auditory verbal learning test; SE: Standard error; CI: Confidence intervals. Mean values were estimated using a linear mixed-effects model adjusted for baseline values and gender. See Methods section for outcome definitions.

**Table S2. Estimated mean and change from the baseline (90% and 95% confidence intervals) in neuropsychological outcomes (other than memory-related) at 3-months and 9-months**

|  | **Art therapy ( N = 22 )** | **Music reminiscence activities ( N = 24 )** | **Control ( N = 22 )** |
| --- | --- | --- | --- |
| WAIS-III Digit Span (Forward) Z-score |  |  |  |
| Mean at baseline (SE) | 1.67 (0.2) | 1.77 (0.22) | 1.85 (0.23) |
| Mean at 3 months (SE) | 2.30 (0.24) | 2.19 (0.22) | 1.48 (0.25) |
| Mean at 9 months (SE) | 2.36 (0.25) | 1.62 (0.23) | 2.08 (0.24) |
| Mean change at 3 months from baseline (SE) | 0.62 (0.31) | 0.42 (0.29) | -0.37 (0.32) |
| Difference [Intervention – Control] (90% CI) [*p*] | 0.99 ( 0.25, 1.73) [0.028] | 0.79 ( 0.07, 1.50) [0.070] |  |
| 95% CI of difference | (0.11, 1.87) | (-0.07, 1.64) |  |
| Mean change at 9 months from baseline (SE) | 0.69 (0.29) | -0.15 (0.27) | 0.23 (0.28) |
| Difference [Intervention – Control] (90% CI) [*p*] | 0.46 ( -0.22, 1.13) [0.263] | -0.38 ( -1.03, 0.27) [0.336] |  |
| 95% CI of difference | (-0.35, 1.27) | (-1.15, 0.4) |  |
| WAIS-III Block Design Z-score |  |  |  |
| Mean at baseline (SE) | -0.78 (0.16) | -0.83 (0.16) | -0.70 (0.17) |
| Mean at 3-months (SE) | -0.08 (0.17) | -0.45 (0.16) | -0.45 (0.18) |
| Mean at 9-months (SE) | -0.30 (0.18) | -0.50 (0.16) | -0.38 (0.17) |
| Mean change at 3 months from baseline (SE) | 0.69 (0.21) | 0.39 (0.20) | 0.26 (0.22) |
| Difference [Intervention – Control] (90% CI) [*p*] | 0.44 ( -0.07, 0.94) [0.154] | 0.13 ( -0.36, 0.62) [0.660] |  |
| 95% CI of difference | (-0.16, 1.04) | (-0.45, 0.71) |  |
| Mean change at 9 months from baseline (SE) | 0.47 (0.19) | 0.33 (0.17) | 0.32 (0.18) |
| Difference [Intervention – Control] (90% CI) [*p*] | 0.15 ( -0.28, 0.59) [0.558] | 0.01 ( -0.408, 0.43) [0.973] |  |
| 95% CI of difference | (-0.36, 0.67) | (-0.49, 0.51) |  |
| Color Trails-2 Z-score |  |  |  |
| Mean at baseline (SE) | -0.79 (0.20) | -0.94 (0.19) | -0.86 (0.20) |
| Mean at 3-months (SE) | -0.28 (0.21) | -0.50 (0.19) | -0.46 (0.22) |
| Mean at 9-months (SE) | -0.31 (0.21) | -0.22 (0.20) | -0.10 (0.21) |
| Mean change at 3 months from baseline (SE) | 0.52 (0.28) | 0.43 (0.26) | 0.40 (0.28) |
| Difference [Intervention – Control] (90% CI) [*p*] | 0.11 ( -0.536, 0.766) [0.771] | 0.03 ( -0.596, 0.663) [0.930] |  |
| 95% CI of difference | (-0.662, 0.892) | (-0.718, 0.785) |  |
| Mean change at 9 months from baseline (SE) | 0.49 (0.28) | 0.71 (0.26) | 0.76 (0.27) |
| Difference [Intervention – Control] (90% CI) [*p*] | -0.27 ( -0.912, 0.372) [0.488] | -0.04 ( -0.663, 0.574) [0.906] |  |
| 95% CI of difference | (-1.037, 0.497) | (-0.783, 0.694) |  |

WAIS-III: Wechsler adult intelligence scale – 3rd edition; SE: Standard error; CI: Confidence intervals. Mean values were estimated using a linear mixed-effects model adjusted for baseline values and gender. See Methods section for outcome definitions.

**Table S3. Estimated mean and mean change from baseline (95% confidence interval) in anxiety, depression and sleep quality outcomes at 3-months and 9-months**

|  | **Art therapy ( N = 22 )** | **Music reminiscence activities ( N = 24 )** | **Control ( N = 22 )** |
| --- | --- | --- | --- |
| Geriatric anxiety inventory total score |  |  |  |
| Mean at baseline (SE) | 2.35 (0.52) | 2.39 (0.49) | 2.55 (0.54) |
| Mean at 3 months (SE) | 1.9 (0.54) | 1.41 (0.5) | 1.31 (0.56) |
| Mean at 9 months (SE) | 2.18 (0.56) | 1.64 (0.52) | 2.5 (0.54) |
| Mean change at 3 months from baseline (SE) | -0.45 (0.72) | -0.98 (0.66) | -1.25 (0.75) |
| Difference [Intervention – Control] (95% CI) [*p*] | 0.80 (-1.25, 2.85) [0.444] | 0.27 (-1.71, 2.24) [0.791] |  |
| Mean change at 9 months from baseline (SE) | -0.17 (0.71) | -0.75 (0.65) | -0.05 (0.72) |
| Difference [Intervention – Control] (95% CI) [*p*] | -0.12 (-2.12, 1.88) [0.909] | -0.70 (-2.62, 1.22) [0.474] |  |
| Geriatric depression scale total score |  |  |  |
| Mean at baseline (SE) | 2.38 (0.44) | 2.1 (0.42) | 2.5 (0.47) |
| Mean at 3 months (SE) | 1.62 (0.46) | 1.35 (0.43) | 1.29 (0.48) |
| Mean at 9 months (SE) | 1.32 (0.47) | 0.95 (0.44) | 2.03 (0.47) |
| Mean change at 3 months from baseline (SE) | -0.76 (0.62) | -0.75 (0.57) | -1.22 (0.64) |
| Difference [Intervention – Control] (95% CI) [*p*] | 0.46 (-1.31, 2.22) [0.610] | 0.46 (-1.23, 2.16) [0.590] |  |
| Mean change at 9 months from baseline (SE) | -1.06 (0.62) | -1.15 (0.57) | -0.47 (0.63) |
| Difference [Intervention – Control] (95% CI) [*p*] | -0.59 (-2.34, 1.17) [0.509] | -0.67 (-2.35, 1.01) [0.431] |  |
| Sleep quality visual analog scale |  |  |  |
| Mean at baseline (SE) | 68.01 (3.18) | 68.7 (3.01) | 69.4 (3.43) |
| Mean at 3 months (SE) | 74.95 (3.32) | 66.6 (3.12) | 72.96 (3.43) |
| Mean at 9 months (SE) | 71.45 (3.4) | 73.21 (3.15) | 71.46 (3.43) |
| Mean change at 3 months from baseline (SE) | 6.94 (4.39) | -2.09 (4.09) | 3.56 (4.62) |
| Difference [Intervention – Control] (95% CI) [*p*] | 3.39 (-9.19, 15.96) [0.595] | -5.65 (-17.82, 6.53) [0.361] |  |
| Mean change at 9 months from baseline (SE) | 3.44 (4.17) | 4.51 (3.82) | 2.06 (4.29) |
| Difference [Intervention – Control] (95% CI) [*p*] | 1.39 (-10.47, 13.25) [0.817] | 2.46 (-8.93, 13.85) [0.670] |  |

SE: Standard error; CI: Confidence intervals. Mean values were estimated using a linear mixed-effects model adjusted for baseline values and gender. See Methods section for outcome definitions.

**Table S4. Estimated mean change in telomere length at 3-months and 9-months**

|  | **Art therapy ( N = 22 )** | **Music reminiscence activities ( N = 24 )** | **Control ( N = 22 )** |
| --- | --- | --- | --- |
| Telomere length |  |  |  |
| Mean at baseline (SE) | 5019 (151) | 5075 (150) | 5019 (166) |
| Mean at 3 months (SE) | 5216 (161) | 5034 (152) | NA |
| Mean at 9 months (SE) | 5570 (168) | 5367 (156) | 5593 (174) |
| Mean change at 3 months from baseline (SE) | 197 (197) | -41 (186) | NA |
| Mean change at 9 months from baseline (SE) | 552 (177) | 292 (163) | 573 (182) |
| Difference [Intervention – Control] (95% CI) [*p*] | -22 (-526, 482) [0.932] | -281 (-766, 203) [0.252] |  |

NA: Not applicable - Telomere length was not collected for the control group at 3-months post-baseline. SE: Standard error; CI: Confidence intervals. Mean values were estimated using a linear mixed-effects model adjusted for baseline values and gender. See Methods section for outcome definitions.
